# Key elements of disaster mitigation education in inclusive school setting in the Indonesian context

**DOI:** 10.4102/jamba.v13i1.1159

**Published:** 2021-08-30

**Authors:** Nurul H. Rofiah, Norimune Kawai, Elli Nur Hayati

**Affiliations:** 1 Graduate School for International Development and Cooperation (IDEC), Hiroshima University, Hiroshima, Japan; 2 Department of Primary Education, Faculty of Teacher Training and Education, Ahmad Dahlan University, Yogyakarta, Indonesia; 3 Graduate School of Humanities and Social Sciences, Hiroshima University, Hiroshima, Japan; 4 Department of Psychology, Faculty of Psychology, Ahmad Dahlan University, Yogyakarta, Indonesia

**Keywords:** disaster education, children with special needs, disabilities, inclusive school, mitigation

## Abstract

Children with special needs are one of the most vulnerable groups when disasters occur. They are often excluded from any risk reduction conducted during such situations; therefore, introducing disaster mitigation education at the early stage has numerous benefits. This study aims to explore the critical elements of disaster mitigation education, limiting the scope to primary schools in an inclusive setting in Yogyakarta. A qualitative methodology involving focus group discussions and interviews was applied for in-depth exploration and insight into stakeholders’ perspectives on education. This study identified six key elements of inclusive disaster mitigation education in schools: (1) strong initiative to conduct self-initiated disaster risk reduction (DRR) education for all students; (2) modification of infrastructure and learning environment to accommodate children with special needs and other students; (3) broadening learning methods in DRR; (4) child empowerment and meaningful participation; (5) school management awareness and strategies for conducting DRR; (6) extensive stakeholder involvement within disaster mitigation education. These elements are expected to improve implementation of such programmes, thereby increasing the quality and accessibility of children’s disaster mitigation education, as well as increasing their capacity in the risk reduction process through teacher support.

## Introduction

Schools need to help fulfil, guarantee, and protect children’s rights, as they spend approximately a third of their time there, with learning lasting for approximately 4 to 8 h daily. Between 2009 and 2018, 62 687 schools in Indonesia were exposed to natural disaster risks (Ministry of Education and Culture 2019), as approximately 75% of schools are located in areas prone to these situations (Wulandari et al. 2020). Consequently, disaster-safe schools are crucial for students, particularly those with special needs.

According to the *Indonesian Law* No. 24 of 2007 concerning disaster management, mitigation education is a series of efforts for risk reduction through awareness and capacity building, as well as physical development, while facing such threats. Specifically, the national action plan contained in the *Ministry of National Education circular* No. 70a/MPN/SE/2010 has a vision of realising a culture of safety and preparedness through a decentralised education system; this system is capable of supporting disaster risk through efforts to reduce vulnerability and increase capacity in the sector. This mainstreaming application prioritises the integration of disaster-mitigation materials into various learning activities.

The latest policy regarding the implementation of disaster-safe school programmes, *Ministry of Education and Culture Regulation* No. 33 of 2019, is regulated by the Ministry of Education and Culture. The strategy for organising disaster-safe schools for vulnerable groups is however, absent from the regulation.

As a result of implementing inclusive education policy regulated by the *Ministry of Education and Culture Regulation* No. 70 of 2009, the number of inclusive schools has increased annually (Musanib 2013). Based on the primary education data for 2018, there were a total of 993 000 students and approximately 91 000 children with special needs in Indonesian inclusive schools. These children mostly have visual, hearing, fine motor, gross motor, speech, intellectual, or specific learning difficulties, attention deficit hyperactivity disorder, or emotional disabilities.

Children with special needs are among the most vulnerable groups in the event of a disaster (Boon et al. 2011; Elangovan & Kasi 2014). Various conditions hinder their ability to implement the mitigation process through both knowledge and practice (Quaill, Barker & West 2019). These children do not receive adequate assistance in participating in disaster risk reduction (DRR) activities (Ronoh, Gaillard & Marlowe 2017), and are often excluded from such activities. Further, some children have mobility barriers to being protected and saved (That et al. 2019). For such vulnerable groups, schools can help provide protection and simultaneous knowledge as also skill improvement.

Moreover, inclusive schools need special treatment related to providing awareness of the risk reduction process from an early age as a preventive measure to allow children with special needs to live in disaster-prone areas. Consequently, problems with the potential vulnerability of children with special needs during disasters are poorly understood. Therefore, this study aims to explore the critical elements of disaster mitigation education in an inclusive setting. The results are expected to improve implementation of such programmes, thereby increasing the quality of and children’s accessibility to disaster mitigation education, as well as increasing their capacity in the risk reduction process through teacher support.

## Literature review

### Overview of disasters in Indonesia

Indonesia is vulnerable to earthquakes, tsunamis, volcanic eruptions, and other geological disasters as it is located between 4 plates: two continental plates: the Eurasian Plate and Australian Plate; and two oceanic plates: the Philippine Sea Plate and Pacific Plate. Furthermore, the National Disaster Management Authority has noted that, as of 2017, there were 2862 disasters, of which nearly 99% were hydrometeorological. These include: floods (979), tornadoes (886), landslides (848), forest and land fires (96), drought (19), earthquakes (20), tidal waves and abrasions (11), and volcanic eruptions (3).

A large-scale earthquake struck Yogyakarta on 27 May 2006. The epicentre was located in the Indian Ocean, approximately 33 km south of Bantul Regency, with a strength of 6.3 on the Richter scale. Consequently, 6000 people died, 40 000 people were injured, and thousands more became homeless. It also had a significant impact on the education sector. This can be seen from the data on damages from 1116 schools in Bantul district, including kindergartens, primary, junior, and senior high schools, where 197 were destroyed, 421 were badly damaged, 344 were lightly damaged, and only 154 remained in good condition.

**FIGURE 1:**
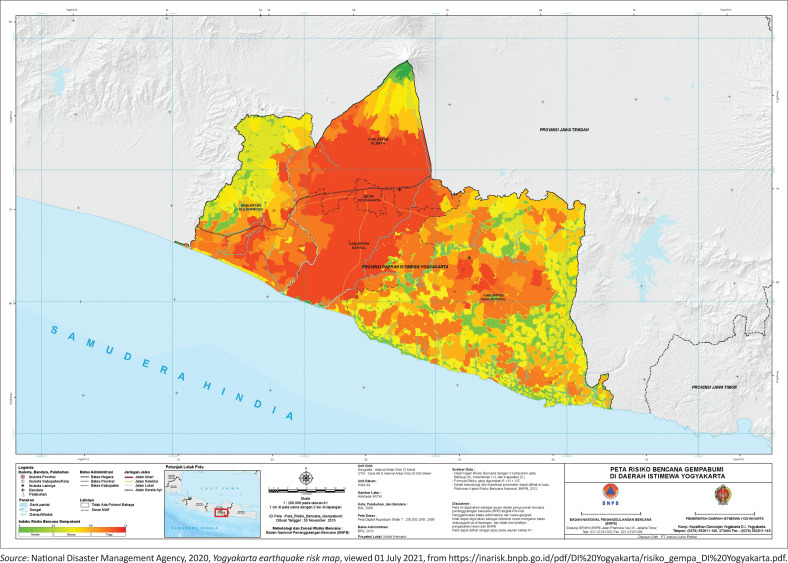
Yogyakarta earthquake risk map.

Yogyakarta is a province susceptible to earthquakes (Figure 1). Geologically, the southern region is the meeting zone of the Eurasian and Indian tectonic plates (Verstappen 2000). Administratively, the location is to the south of Mount Merapi with an area of approximately 3185.80 km and a density of 1201 people per km^2^, and over half the area is dryland. The northern region, namely the Sleman Regency, is prone to volcanic disasters, whereas the Bantul Regency in the south is prone to earthquakes and tsunamis. Based on Yogyakarta Regional Disaster Management Agency (2020), in the rainy season in January 2020, the Yogyakarta area was dominated by landslides (133 events), fires (37 events), strong winds (30 events), and earthquakes (2 events).

The Yogyakarta government has given access to people with special needs as an accelerating step towards building an inclusive area. In this province, inclusive education has been administered in 109 primary schools – 22 in Sleman, 26 in Bantul, 26 in Gunung Kidul, 13 in Kulon Progo, and 22 in Yogyakarta City (Rofiah & Kawai 2019). The focus of our study is Yogyakarta as it is one of the disaster-prone areas in Indonesia as also an area that implements inclusive education.

### Disaster mitigation education

Awareness of disaster mitigation measures to reduce disasters can be created through education channels and increased capacity by applying specific science and technology and using counselling services with simulation techniques (Indriyani 2011). This needs to be carried out through both formal and non-formal education (Rizaldy 2018). The emphasis on disaster mitigation involves awareness and capacity building, as well as physical development in facing the associated threats (Suarmika & Utama 2017). The purpose is to build a system that combines technology engineering with legal, administrative, economic, managerial, and educational aspects to secure development and social stability. The steps taken include developing scientific studies and utilising modern technology to create mitigation mechanisms following local conditions (Setiawan 2010; Susanto & Putranto 2016; Wibawa, Citra & Tika 2013).

One mitigation effort that is often applied involves establishing a disaster-safe school that requires three main pillars: safe learning facilities, school disaster management, and mitigation education (Ministry of Education and Culture 2015; UNISDR & Global Alliance for Disaster Risk Reduction & Resilience in the Education Sector 2017; World Bank Disaster Risk Management 2017). Furthermore, the framework establishes essential principles that consider people with special needs. These policy guidelines and principles advocate for a two-track approach to risk reduction projects, including comprehensive accessibility, universal building design, non-discrimination, coordination, and collaboration across all DRR activities.

## Method

A qualitative methodology involving focus group discussions (FGDs) and interviews was applied to explore stakeholder perspectives on disaster mitigation education in an inclusive school setting. FGDs provide qualitative study teams with the ability to systematically and concurrently question multiple respondents (Boateng 2012). This was conducted with each of the nine participant groups (Table 1): (1) one for education authorities, (2) one for the regional disaster management agency, (3) one for a non-governmental organisation (NGO), (4) one for principals, (5) one for teachers, (6) two for school committees, and (7) two for student groups, including children with special needs in primary schools. The student participants recruited were children between 9 and 11 years of age. The main objective of the FGDs was to gain insights into stakeholders’ perceptions of their disaster preparedness in an inclusive setting.

**TABLE 1:** List of participants.

Participant groups	Number of participants
Education authorities Yogyakarta	2
Regional disaster management agency Yogyakarta	2
Non-governmental organisation	2
Primary school principals in an inclusive setting	14
Primary school teachers in an inclusive setting	20
School committee (female and male)	10
Student group including children with special needs	20

The collected data were investigated using thematic analysis (Braun & Clarke 2006) according to the outlined five-step methodology: (1) begin by reading and rereading the original transcript; (2) create the code by labelling the fragments verbatim; (3) identify the codes that appear to be fundamental to the study, then group them into themes and sub-themes based on the patterns of meaning developed between them; (4) assess the themes or sub-themes, as well as any possible connections between the codes, sub-themes, and themes; and (5) prepare a list of the results and insights and connect them to the inquiry to create a compelling and coherent story.

Owing to the COVID-19 pandemic, FGDs and interviews were implemented in two ways: face-to-face (in person) and using virtual platforms. Focus group discussions with parents and NGOs were conducted using the WhatsApp application (video call group), with which the participants were familiar. Meanwhile, FGDs with education authorities, regional disaster management agencies, principals, teachers, and student groups were conducted in person. All collected data were recorded with the respondents’ consent and transcribed to facilitate the analysis process.

## Results and discussion

**FIGURE 2:**
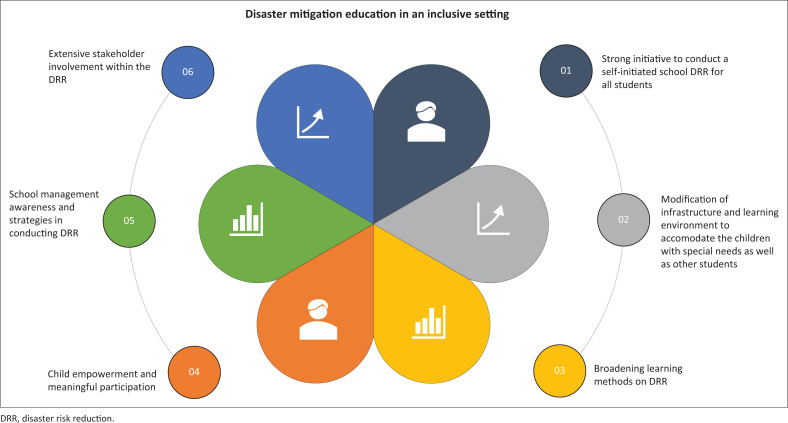
Key elements of disaster mitigation education in an inclusive setting.

This study aimed to explore the critical elements of inclusive disaster mitigation education in schools. When the transcripts were read several times, a ‘sense’ of the data was obtained, followed by creating an image. Afterwards, significant phrases, sentences, or statements were coded for the purpose of this study, while the meaning of the identified units was grasped by reformulating them (see Figure 2). The descriptively formulated meanings were interpreted in the reflective section of the analysis, and sub-themes were identified. Finally, the sub-themes were grouped into themes and combined into a comprehensive phenomenon (see Figure 2). The following results and discussion provide a summary of disaster mitigation education activities in schools. The six themes generated were as follows:

1. Strong initiative to conduct self-initiated DRR education for all students

Self-initiated DRR is a crucial aspect of implementing disaster mitigation education. Therefore, awareness of all school members is necessary when preparing to face disaster risks and minimise the number of victims:

‘At the beginning of 2006, in the Bantul area, there were frequent large earthquakes. Then, we took the initiative so that if an earthquake occurred in school, the risk could be reduced; thus, children were trained on what to do in case of an earthquake. Teachers were also trained at first; simulations were used for this purpose.’ (Senior teacher, male, 54 years)

‘Our school carries out disaster education independently. Initially, we collaborated with the KYPA [*Komite Yogyakarta untuk Pemulihan Aceh*] (NGO in the field of disasters). Our training was performed independently. The school did not receive funding from anywhere. Schools are committed to the importance of implementing disaster education. Teachers, students, and parents should be aware that disasters can come anytime and anywhere.’ (Principal, female, 48 years)

Schools A and B in the Bantul district explained that they were busy completing the integrated programme content. Generally, schools have limited time to implement DRR education, coupled with a lack of financial support from the government. Therefore, their awareness of this activity is important. A study on implementing disaster preparedness education in New Zealand primary schools discovered that one deterring factor that was often encountered was the lack of time to incorporate disaster-related subjects into classroom activities (Johnson et al. 2014). Amri et al. (2017) reported that two of the most important issues in the implementation process were personnel dedication and budget, while lack of funding also influenced DRR.

The school initiatives in implementing inclusive DRR involved teachers identifying children with special needs individually, followed by planning corresponding learning. As mentioned by the education authorities, teachers have to understand children’s learning profiles. This was carried out by recording the children’s diversity, special tools needed, accessibility needs for mobility, abilities, strengths, and information about diagnosis and treatment to complete their health history.

Education authorities of Yogyakarta state:

‘The student learning profile instrument aims to help teachers better understand the difficulties and needs of individual students. This instrument will be developed into an application that will be linked to the Ministry of Education and Culture’s Basic Education Data.’ (Education authority, male, 48 years old)

Several schools stated that they still found it difficult to successfully identify children with special needs. According to them, special skills are needed for this purpose, such as those possessed by psychologists or paediatricians. Training for teachers is indispensable in identifying and assessing children with special needs, while the data are used in fulfilling needs, participation, capacity building, and appropriate and specific protection priorities (Sloman & Margaretha 2018).

2. Modification of infrastructure and learning environment to accommodate children with special needs and other students

Modification of infrastructure and learning environment ensures that they become accessible to children with special needs, based on the intention of opening up opportunities, enabling independent living, and full participation in all aspects of life. Accessibility is implemented using universal design principles and reasonable accommodation to provide comprehensive information in an accessible format, namely, learning materials, communication media, and early warning systems:

‘For the facilities, you can see the building; even though children are good at dealing with earthquakes, it is useless if the building is not disaster-safe. The building must be sturdy, and the two doors must open outwards if opening inwards is difficult. The table edges are deliberately blunted to reduce the risk, as well as other facilities that are tailored for child safety.’ (Teacher, male, 30 years)

Children with special needs are required to utilise all public facilities, including schools. Hence, physical development is required for everyone to easily access the environment. The ease of mobility is related to physical development, such as ramp construction for wheelchair users, guide lane installation for the visually impaired, and accessible pedestrian pathways and toilets (Margaretha 2020). As an education service provider operating in an integrated manner, an inclusive education organising school is essential for creating accessibility to daily activities and easy evacuation during a disaster. However, when the evacuation route is not accessible, the children are at risk.

According to *Indonesian Law* No. 28 of 2002 concerning buildings, accessibility is the facility provided for all people, including those with special needs and the elderly, to realise equal opportunities in all aspects of life. It enables everyone to perform their activities safely, efficiently, and independently, without discrimination. In this sense, there are two types of accessibility: non-physical, meaning the ease provided to everyone to enter, use, and exit a system, and physical, which is a convenience provided to carry out the same activity.

Mapping educational activities in various disaster-prone areas in Indonesia and supporting capacity building for education remain minimal. Moreover, a study conducted in various regions showed a lower level of preparedness in schools than the community (King et al. 2019). For vulnerable groups such as children with special needs, schools can help provide protection and simultaneous knowledge and skill improvement (Mcdermott, Martin & Gardner 2016), especially those in an inclusive setting (Sloman & Margaretha 2018). Disaster mitigation is closely linked to risk reduction activities before the disaster stage (Stough & Kang 2015), which are crucial because they are useful during and after the disaster (Peek & Stough 2010).

3. Broadening learning methods on DRR

Delivering information about disaster mitigation for children with special needs requires different methods. For example, those suffering from hearing impairments require different ways of absorbing information. Such children have limited memory, struggle to complete complex tasks independently, are more distracted/forgettable, and require more directions than others. Modification and accommodation of DRR education for these children is critical. However, thus far, the material on DRR that has been prepared to meet their needs is minimal.

Teachers used concrete media to facilitate DRR education, and approximately an equal number of older and younger groups of students were discovered to have been using FM radio and television to obtain disaster information. However, the younger ones were more interested in newspapers than their older counterparts (Tuladhar et al. 2014).

A female student said:

‘I mostly like learning by doing. By practising directly, it makes it easier for me to understand and remember what to do during a disaster. Sometimes learning theory is very boring, and I forget a lot.’ (Student, female, 11 years old, 5th grade)

The lesson plan began by mapping the core and basic competencies contained in each theme. Teachers integrated disaster mitigation knowledge into teaching and learning resources. They no longer relied on textbooks but used other learning sources such as local wisdom, using which many values were taught. In addition, fun learning methods have been employed, such as utilising songs in DRR education.

‘There is a kind of song so that our children will always remember. We teach the children that (song) so that they will remember.’ (Senior teacher, male, 55 years old)

Teachers prepare an individual education program (IEP) for children with special needs while working with the remaining IEP team to plan for their safety, even during a national lockdown or in the event of a natural disaster. The IEP team understands the intellectual, physical, emotional, and health needs of such children; therefore, it is best suited for designing and realising an individual emergency lockdown plan (Clarke et al. 2014; Dusty et al. 2019).

The results showed that the major problem in implementing mitigation education in schools is a lack of understanding among teachers and students about DRR and management, as also observed by Perwira (2016). Furthermore, the outreach results in the form of training, seminars, or field rehearsals showed different levels of teacher understanding of the socialisation material (Perwira 2016), which affected their teaching and learning activities. Many students were confused during the training because of their different understandings. Another problem is the lack of teachers’ capacity and expertise in integrating DRR into the curriculum (Wedyawati, Lisa & Selimayati 2017). Therefore, training teachers and students in DRR education are essential for statistically effective improvement. A positive relationship was discovered between the demographic variables of teachers’ knowledge and disaster management practices (Abozeed et al. 2019).

Besides the lack of teachers’ preparation and education in the management process, another major problem was the occasional use of experience-based, immersive, and action-oriented learning activities (Apronti et al. 2015). Often, board games, disaster storybooks, poems, and songs are not utilised, particularly in developing countries where technology is not easily accessible. Raising awareness and passing theoretical knowledge on disaster prevention and response is insufficient. However, students need to be equipped with appropriate skills and competencies.

4. Child empowerment and meaningful participation

The lead resource identified several questions to learn about the staff’s level of understanding in the sample schools; this was based on information gained on which person needs to be safeguarded and how this can be done, as well as their overall roles and responsibilities during and after disasters. The more disabled people understand and manage their risk during disasters, the less vulnerable they are. The key to this process is empowerment, which is directly linked to the identification and understanding of these groups (Bennett 2020). Empowerment is understood as a cognitive transfer issue, but the interest and motivation of children in schools are also changing.

Schools promote the continuous involvement of children with special needs in DRR planning and disaster simulations or drills through targeted and effective participatory tools (e.g. maps) to help them feel included and become informed. Supported people create individual disaster plans within a school context. Such an approach requires teachers to be an important bridge to inclusiveness in the DRR programme. During the involvement, the two most essential principles were, first, the importance of providing safe spaces for children to process their experiences and, second, the belief in having the right to participate in decision-making that affects them (Mutch & Gawith 2014).

Schools need to ensure the implementation of non-discriminatory disaster mitigation education. Policies or regulations support children with special needs to increase their capacity. Further, they can be involved in DRR planning, disaster risk assessment, and decision-making while attending school; they are also allowed to become facilitators in DRR.

5. School management awareness and strategies for conducting DRR

Disaster management in schools is an assessment process that continues with planning for physical protection, capacity building in carrying out emergency responses, and education continuity at the respective school level up to all education offices, namely, district or city, as well as provincial to national levels. Based on the results, a number of strategies were identified for each of the following stages: preparation, planning, and sustainability (see Table 2).

**TABLE 2:** School management awareness and strategies for conducting disaster risk reduction.

Preparation	Planning	Sustainability
Strategy for establishing a school disaster management committee.	Preparation of permanent procedures at school.	An independent DRR initiative that is carried out continuously.
The existence of policies, agreements, and/or school regulations supporting efforts to reduce disaster risk in schools.	Complete standard disaster mitigation equipment.	Regular participatory monitoring and evaluation of school readiness and safety (regular school preparedness testing or training).
-	The early warning system is adapted to the conditions of all children. Hence, disaster warnings become accessible to everyone.	-

DRR, disaster risk reduction.

In the preparation stage, most schools in the four sample regions did not have emergency handling committees because they were not equipped with the requisite funding to allow their emergency handling committee to use the necessary expertise and skills for providing leadership in times of crisis. This absence of emergency management committees posed severe threats to students and teachers. However, some schools had emergency management committees that played the lead role in developing sound prevention and preparedness systems to minimise disaster impacts and handle such situations (Shah et al. 2020). Meanwhile, regarding the inclusive DRR policy, all schools reported that this had not been explicitly observed in their vision and mission. However, School C carried out DRR education independently because it was located in a disaster-prone area. The establishment of the aforementioned Disaster Management Committee is more powerful and meaningful when supported by the existence of policies or regulations backing DRR efforts in schools.

Three important aspects are required in the planning process: developing permanent disaster preparedness procedures, providing disaster mitigation equipment, and disseminating an early warning system accessible to all school residents, including children with special needs. The principal stated:

‘Our school prepares standard operating procedures assisted by KYPA (NGO), such as procedures for independent evacuation from buildings, procedures for evacuation to safe places, and so on. Yes, we will do it in stages. Everything is communicated to all members of the school, including the parents of students.’ (Principal, female, 46 years old)

Based on the interview results with the teacher, schools do not have an accessible early warning system for children with special needs. So far, schools use *kentongan* (which is a communication tool made of bamboo or wood deliberately hit to provide information to others) as an early warning sign. However, teachers are aware of the need for an early warning system for children with special needs and disabilities. A teacher at School C stated:

‘Schools should have an early warning system that can be accessed by everyone, for example, for children with hearing or visual disabilities. Safety is everyone’s right, and schools should facilitate it.’ (Teacher, male, 28 years old)

At the sustainability stage, there were two important aspects: ensuring the continuous implementation of disaster education and regularly evaluating school preparedness and safety. Disaster risk reduction education was carried out during training from the Badan Penanggulangan Bencana Daerah (Regional Disaster Management Agency) or other NGOs, and there has been no evaluation of the programme’s sustainability in schools, except for those with personal initiatives.

6. Extensive stakeholder involvement within disaster mitigation education

Schools need to collaborate with relevant stakeholders (e.g. local health officials and the Red Cross) based on the aim of supplementing DRR messages, such as ‘drop, cover, and hold’ and options for children with special needs, for example, those using wheelchairs and those with visual or hearing impairments. Students tend to share information with their families and the community on disaster preparedness and ensure that no one is left behind during a crisis; further, the outcomes are potentially improved by partnering with families.

A senior principal stated:

‘We cooperate with parties outside the school. This strategy revived the *gotong royong* (mutual cooperation) from the beginning. We also take advantage of *Karang Taruna* (the social youth organisations in the village or sub-district area) by starting to create an evacuation route. The teachers must also efficiently support each other when there is low enthusiasm. The sustainability of the program involves involving residents around the school in disaster simulations.’ (Principal, male, 49 years old)

This was supported by parents at school A:

‘We are members of the student guardian association. Parents are involved in disaster simulations at schools. We have regular meetings to get information about disaster risk reduction.’ (Parent, female, 39 years old)

Pertiwi et al. (2019) discovered two possible approaches for ensuring cooperation among multiple stakeholders. The first was conventional players in disaster risk mitigation reaching out to disabled people organisations (DPOs). This appeared to be the aim of the Sendai Framework, which was supported by Indonesian legislation. The other option was for DPOs to become acquainted with disaster risk mitigation governance and stakeholders in their country. Considering this, school authorities in Yogyakarta province need to conduct regular meetings to discuss possible crises and their willingness to share resources with neighbouring colleges, state agencies, and community organisations.

## Conclusion

Six key elements of implementing inclusive disaster mitigation education in Indonesian schools were identified based on stakeholders’ perspectives: (1) strong initiative to conduct self-initiated DRR education for all students; (2) modification of infrastructure and learning environment to accommodate children with special needs and other students; (3) broadening learning methods in DRR; (4) child empowerment and meaningful participation; (5) school management awareness and strategies for conducting DRR; and (6) extensive stakeholder involvement within disaster mitigation education. The diversity of children with special needs and disabilities requires various adjustments to realise school’s disaster preparedness. Therefore, the identification of inclusive disaster mitigation education tends to increase the capacity of children with special needs in DRR. Additionally, this study acknowledges the limitations of its methodology and the small sample size of only one province. Suggested areas for further research include determining the impact of integrating hazard and disaster topics in the school learning programmes in inclusive settings and investigating the effectiveness of disaster education programmes for children with special needs and disabilities.
